# How the Built Environment Promotes Residents’ Physical Activity: The Importance of a Holistic People-Centered Perspective

**DOI:** 10.3390/ijerph19095595

**Published:** 2022-05-05

**Authors:** Yufang Zhang, Terry van Dijk, Cor Wagenaar

**Affiliations:** 1 Department of History of Architecture and Urbanism & Expertise Centre Architecture, Urbanism and Health, Faculty of Arts, University of Groningen, P.O. Box 716, 9700 AS Groningen, The Netherlands; yufang.zhang@rug.nl; 2 Department of Spatial Planning & Environment, Faculty of Spatial Sciences, University of Groningen, P.O. Box 800, 9700 AV Groningen, The Netherlands; t.van.dijk@rug.nl; 3 Faculty of Architecture and the Built Environment, Delft University of Technology, P.O. Box 5, 2600 AA Delft, The Netherlands

**Keywords:** built environment, physical activity, urban analysis, walking and cycling, neighborhood design, China

## Abstract

Promoting adequate physical activity (PA) such as walking and cycling is essential to cope with the global health challenge of non-communicable diseases (NCDs). Much research has been conducted to analyze how the built environment can promote PA, but the results are not consistent. Some scholars found that certain built environments such as green spaces generated positive impacts on PA, while some other studies showed no correlations. We suspected that the built environment should be measured in a deeply holistic nuanced way in order to properly reflect its impact on PA. Therefore, our research adopted an integral urban-analysis comparing three typical neighborhoods in Beijing, China. Our data show that the highest PA occurs in the neighborhood with the lowest density, amount of green space and street connectivity, apparently compensated by its low-rise housing type and high appreciation of the quality of sidewalks and street safety. This indicates that dimensions impacting PA have to be considered in context, and the peoples’ perception of the built environment matters.

## 1. Introduction

Public health in cities is a crucial topic, especially with trends of rapid urbanization. In 2020, over 55% of the world’s population lived in cities, and it is expected to increase to 68% by 2050 [1,2]. Unhealthy lifestyles have emerged among citizens, such as physical inactivity, thus resulted in an increasing prevalence of many kinds of non-communicable diseases (NCDs), such as diabetes [3,4].

**Figure 1:**
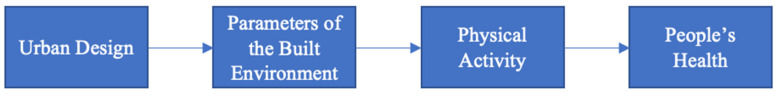
The causal chain from urban design to health.

To improve public health, many efforts have been made to shape healthy lifestyles for citizens, and urban design (which directly changes the human-made built environment) has played an essential role [5,6]. For example, the ‘Heathy Cities’ movement, initiated by the World Health Organization and well received worldwide [7,8], has acknowledged the importance of improving the built environment through urban design, as a way to support citizens in performing a healthy lifestyle. The assumption in the policy effort and the studies that promoted them are shown in the causal chain (Figure 1): when people live more active lives, their general health will increase. As people’s living environment can either facilitate or block such a lifestyle, good urban design of the built environment (BE) can make people healthier by affecting their physical activity (PA).

Part of the causal chain, i.e., the impacts of the built environment on physical activity, has aroused great attention in the last two decades [9,10,11,12]. The built environment is broadly interpreted as the human-made spaces in which people live, work, and play on a day-to-day basis, such as residential buildings, streets and sidewalks, open spaces, etc. [13]. The term physical activity refers to all movement, not only sports and performing active recreation activities such as yoga and tai chi, but also daily walking and/or cycling [14,15], occurring for a variety of utilitarian motives. 

Statistically positive relationships between the built environment and physical activity, just as assumed in the Healthy Cities movement, have been scientifically demonstrated (Table 1). For example, Song et al. [16] found that some built environment characteristics, such as access to parks and playgrounds and neighborhood safety, appear to promote daily mobility by walking or cycling, as well as recreational physical activity among older adults in Singapore. A longitudinal study performed in the UK showed that physical activity can be enhanced by increasing people’s proximity to nearest parks and public transport stops [17]. Moreover, a systematic review [18] found five built environment aspects that are positively related to increased physical activity levels: diversity in housing types, mixed land use, housing density, compact development patterns, and amount of open space.

However, the results are not always consistent. In a study based in the Australian context, built environment attributes such as lower residential density, lower street connectivity, less land-use diversity, and poorer access to public transport were found positively related to higher levels of physical activity [19]. This finding runs in the opposite direction of many other studies. Similarly, the presence of green space was identified to be positively related to walking by Astell-Burt et al. [20], while no significant relationship was found in a study conducted in the Netherlands by Zhang et al. [21]. More contradictory evidence will be discussed in the next Section. 

It is for this reason that we add evidence to the causal chain between BE and PA by using a holistic human-centered approach, which sees places as an integrated complex system with people and other important stakeholders at the center of the design and implementation process [22,23], to assess the built environment and then explore its linkage to physical activity. We chose neighborhoods which are representative for a neighborhood type (thus reflecting a variety of urban designs), and assess their built environments to reveal how built environments impact physical activity in a nuanced way. 

Our study reveals how *combinations* of built environment parameters impact physical activity in neighborhoods, together with the perceived quality of these features. The paper is structured as follows. The review in Section 2 takes stock of the features assumed by the literature to influence physical activity. The exiting evidence is explored and assumptions are posed on why the complexity in interdependent parameters deserves more scrutiny. Section 3 explains our research design, in which we combine spatial analysis and self-reported activity patterns and health to compare three neighborhoods in Beijing. The neighborhoods are introduced in Section 4. Findings are presented in Section 5. The discussion in Section 6 discusses what the findings mean for how best to understand the physical activity levels as a consequence of the urban design of neighborhoods, providing new insights for making cities healthier.

## 2. Literature Review—The Missing Part in Assessing the Built Environment

Five aspects of the built environment have been frequently explored and found to impact physical activity: density [24,25], land-use diversity [24,25,26,27,28], street connectivity [16,28], as well as the availability of urban green space [20,21,29] and public transportation [16,17,28]. These all affect a person’s decision to leave his home by foot or by bike instead of a car, for either leisure, shopping, exercise, or work. Other likely incentives to discourage people from either having a car or parking one’s car close to home include the nature of the climate and cultural factors, but these do not fall under the scope of this paper. 

The five extensively studied parameters of the built environment have been assessed in a variety of ways, but the outcomes have not been entirely consistent. We suspect this may be the result of two recurring features in this literature: (1) parameters are usually treated separately, and (2) parameters are operationalized using generic spatial data sets that overlook people’s perceived realities. Although we cannot here review the entire body of knowledge, we do want to illustrate why a holistic and human-centered approach is highly relevant.

As for problem (1), the separate treatment of parameters is found in studies [20,29,30]. These researchers concentrate on a specific parameter (e.g., green space), ignoring the fact that each parameter is, in reality, factored into the complex personal considerations of individual urbanites on whether or not to be physically active outdoors. Although every parameter in itself can have an enabling effect, in reality multiple factors need to be favorable at the same time to enable residents to choose active modes of mobility through the neighborhood. This may explain why some data sets of parameters do not show that they have expected influences on physical activity.

Although some studies simultaneously include multiple parameters [19,28], they generally do not approach these in a holistic way. Correlations between the respective parameters and physical activity are sought separately. 

As for the other problem (2): generic data sets used to calculate parameters, we see many studies that use readily available GIS data to estimate the parameters under investigation. With generic data sets, we mean the land use maps, road maps, building footprints and heights, that can be obtained at geo-information firms, such as Esri. Although these sets are of value as a basis to be charged with more detailed data, the use of generic data is tempting and these parameters require a much more subtle and nuanced analysis that reflects the complex way of how the aspects of the built environment influence people’s PA than a GIS layer or calculations suggests. Regarding something as seemingly straightforward as the residential density of an area, some studies calculate this parameter from available GIS data sets on home occupancy and housing units per hectare [16,24,31,32]. However, the PA of individuals is likely to differ widely between an area where all people on that hectare live in a few high-rises with shops in every basement (as is common in many Asian mega cities) and an area packed with many small single-story houses with shops in a few central streets—even though their calculated densities may be identical. 

To measure land-use diversity, an entropy index has often been used to quantify the degree of mixing across land-use categories within a studied area in the GIS system [28,32]; each land-use category was given the same value. However, in actual use, the various destinations can be valued very differently by the users, strongly impacting behavior.

Similarly generic is the treatment of street connectivity in the available studies. Although actual PA depends highly on a fine-grained mesh of alleys and paths that are experienced as comfortable and safe, some studies translate street connectivity into the number of intersections relative to the analyzed area [28,32,33]. However, which roads are included in the GIS model, and which smaller walking routes are invisible? Further, what quality differences in the walkability of any given road section are here ignored?

Generic calculation of green space availability is also problematic. Green space is often measured by the number of square meters on GIS maps or remote sensing pictures, and thus defined as green space without differentiating its various qualitative features [20,28]. Without assessing the quality of the green (or the amenities provided), its physical existence and proximity to people’s houses is hard to link to PA. The same problem exists in the availability of public transit: to count only the number of transits without noting their distribution or quality of service can reveal only a part of the relationship.

Aggregated neighborhood measures are also used to assess the built environment. The walkability index (usually combining land-use mix, residential density, and street connectivity, based on GIS data) was found to be positively related to PA [17,31,32,33,34]. In the Australian context, convincing evidence also indicated the positive impact of walkability on PA [35]. Perceived built environment has aroused attention as well [36,37], and results showed it is a necessary complement to the objectively measured built environment. Awareness of the role of esthetics and safety [12,38] has also been growing (often assessed with self-reported data). They were tested to be relevant as well, but the results varied across studies. 

We suspect that when the density, street pattern, green space quality, etc. are not operationalized in the right way, and are not combined with other activity-enhancing conditions, the evidence for the expected effect (activity and thus health) will be inconsistent. Results regarding experienced safety, for example, seem to negate the general assumption that safer neighborhoods generate more physical activity. Additionally, the analyses in the literature mentioned here presupposed that the availability of the environmental features they address were not compromised in any way (by fences, parked cars, lack of maintenance, decay, etc.), but in real life, quality and accessibility may differ radically from the model. Our study, therefore, evaluates a neighborhood as an integral entity, which means besides using the largely used parameters (e.g., density, land use diversity, street connectivity) to describe the physical environment, we also look into its history, the design strategies behind its current spatial patterns, and resident perception of the neighborhood in order to provide an encompassing image of a neighborhood (Table 1). Holistic urban analysis in neighborhoods takes neighborhoods as a complex entity, and explores how the built environment affects the level of physical activity of its residents.

We explore how to best understand people’s proclivity to physical activity in neighborhoods by empirically testing the validity of the following assumptions: 

(1) High population density, land-use mix, street connectivity, abundance of green space, and availability of public transit availability form a complex of mutually compensating or cancelling parameters that should be considered in concert in order to understand physical activity levels.

(2) Combining perceived measurements and observational measurements can better capture the built environment features (both quantity and quality) that determine an individual’s behavior, because calculated parameters can conceal nuances that are crucial for people’s actual behavior.

## 3. Method

Testing the above hypotheses requires a comparison of physical activity in a set of neighborhoods that have maximum variety in the nature of their built environment, but are as similar as possible in other factors known to influence lifestyle and health.

Similarity in the other health-related factors can best be determined by comparing neighborhoods within one city, where people are exposed to similar air quality, culture-related stress levels, habits, food products, health care and education systems. Additionally, people’s socio-economic status (education, income, age) must be similar while the spatial characteristics differ. Neighborhood data are required to confirm whether non-spatial health determining factors were indeed comparable. 

**Figure 2:**
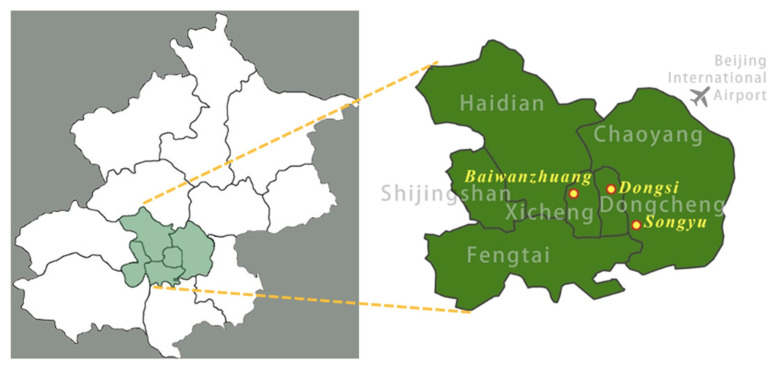
The map of Beijing and the location of three neighborhoods (by author).

We selected three neighborhoods in Beijing, China, that provided large homogeneous units differing widely in built environment characteristics but similar in other health-defining factors. The three neighborhoods were located in central Beijing, namely Dongsi, Baiwanzhuang, and Songyu (see Figure 2). Multiple research methods were adopted, including document analysis, spatial analysis, field observation, and questionnaire survey.

For this analysis, the daily lives of the people whose activity data were collected must be influenced by the characteristics of their neighborhood. Therefore, we approached people who are surrounded by a relatively large homogeneous area of a certain type of built environment. Homogeneity in a spatial radius of at least 300 m is key; otherwise, people’s health and physical activity could be impacted by features that are available nearby but outside the neighborhood. 

We conducted a document analysis to learn about the development of neighborhood planning theories in Beijing, as well as the background, history and residents’ SES of the three selected neighborhoods [39,40,41]. Then, we applied the urban analysis and field observation to assess the built environment of the three neighborhoods (A). The built environment dimensions which have been widely measured in the literature (density, land-use mix, street connectivity, green ratio, public transit availability) were chosen but assessed in a more nuanced way, to map out people’s real-use experience of the spaces and facilities in their living neighborhoods. Due to the lack of digital spatial data sets on the neighborhood level in Beijing, the urban analyses were mapped by hand with the use of the basic Baidu Maps that was filled with field observations. The filed observation was conducted in March 2021 by the primary author. Sidewalks and green areas within the neighborhoods were categorized into ‘poor quality’, ‘fair quality’, and ‘inaccessible’ for reasons of their general condition (such as holes on the sidewalk), parked cars blocking the way, and equipped facilities [42,43]. The results are shown in the urban analysis maps.

The data on the amount of residents’ physical activity (B) and perceptions (C) were collected by means of a questionnaire. We designed and tested the questionnaire with residents first (in February and March 2021), and then conducted the survey in March and April 2021. The primary author and three other trained investigators randomly distributed the questionnaires in the center area of Dongsi, Baiwanzhuang, and Songyu neighborhoods, to ensure the representativeness of the residents and most impacts are from their living neighborhoods. In the latter two neighborhoods, local communities assisted with the distribution. 

There were three main sections in the questionnaire: (1) general information on the interviewees, such as age, gender, net household income, education level; (2) residents’ physical activities (walking, cycling, etc.) in their living neighborhood in a general week (other PA such as walking at work was asked not to be counted); the behaviors were categorized by the motivation, such as going to work, going shopping, and going for recreation. This section was revised based on the International Physical Activity Questionnaire (IPAQ), which has been validated and broadly applied in empirical studies [44]. Residents were then asked about (3) their perception of their living environment in terms of its safety, aesthetics, and people’s satisfaction with the green spaces and sidewalks. We also asked, ‘Do you want to move to another neighborhood in the following six months?’ to avoid self-selection bias [45]. A 5-point Likert scale was employed. People who are between 18 and 65 years old, have no disabilities that would affect walking/cycling, and have lived in the neighborhood for more than 6 months were invited to participate.

After obtaining the data, we compared physical activity levels in three neighborhoods, and examined if the result was consistent with the expected parameters that are common in previous built environment studies (this will be presented and discussed in the Section 5 and Section 6, using Table 6). We combined the parameters and physical activity levels with the calculated, observed, and perceived qualities of the built environment. We searched for counterintuitive combinations of parameters, validating or denying our two assumptions rather than formal statistical correlation, due to the focus of the neighborhood entity of this study.

## 4. Introduction to the Three Neighborhoods

Neighborhood design in China has gone through several phases. Since the 1940s, Chinese neighborhood planning strategies have shifted under the significant impact of soviet and western planning ideas [41]. Economic development is another impact factor that has brought changes in planning strategies [41]. We categorized the neighborhoods into four typologies according to different planning strategies (Table 2). Our study cases represent the first three neighborhood types.

### 4.1. Dongsi

Dongsi is a typical Beijing Hutong neighborhood (built in the 1300s and later), located in Dongcheng district, the center area of Beijing. The whole area is 0.87 km^2^, and the population is around 13,000 (since the available documented data is not detailed for the neighborhood level, the number of areas and population have been obtained using the search engine Baidu; the same applies to the population data below). The buildings in the neighborhood are mostly Siheyuan—a common historical type of residence in Beijing. The Siheyuan is a one-floor building with a yard surrounded by rooms, and used to be regarded as luxury housing due to its spaciousness. However, nowadays in Doingsi, one Siheyuan could be shared by several, or even more than 10, households. The Siheyuan buildings were mostly built between the 13th and 19th centuries. After the 1950s, some other buildings were built with more floors for housing or commercial use. The residents are mainly local Beijingers, who have lived there almost all their lives. The environment is always perceived as quiet, but the downside is that there are nearly no green areas.

### 4.2. Baiwanzhuang

After the Second World War, Western planning theories were introduced to China, making the Chinese planning field more different than ever before. Chinese neighborhood design from the 1950s first borrowed the idea of the neighborhood unit from Clarence Perry, and was later widely impacted by neighborhood area concepts imported from the Soviet Union [41]. The central idea of the neighborhood area is to create a community-centric lifestyle. Baiwanzhuang is the first neighborhood built in China based on the philosophy of neighborhood areas and with the help of Soviet experts. The last few neighborhoods built in the 1950s in Beijing are there today. 

Baiwanzhuang is located in the center of Beijing, in the Xicheng district. The buildings in the north were built mainly in the 1950s and 1960s, and the buildings in the south were built in different years (from the 1950s onwards). The ones built in the 1950s are mainly three-floor buildings; later built buildings are usually higher. The total area of Baiwanzhuang is 1.02 km^2^, inhabited by around 31,000 residents, most of whom are from Beijing.

### 4.3. Songyu

Songyu neighborhood was built in the 1990s. It is a gated neighborhood located in the Chaoyang district. Six-floor residential buildings and twenty-floor high-rise buildings stand in the neighborhood. The total area is 1.05 km^2^, inhabited by around 35,000 residents. Following the planning strategy of that time, Songyu was designed with relatively more green and public spaces: there is a public park in the center of the neighborhood, several medium-size green and public areas distributed throughout, and many pocket green spaces in between the buildings. There are diverse shops and other facilities.

### 4.4. Similarity in the Three Neighborhoods 

To indicate the social features in the neighborhoods studied, we used the data at sub-district level because of the similarity of neighborhoods in one sub-district, and the lack of data at the neighborhood level (Beijing has 16 districts; dozens of sub-districts belong to these districts, while several neighborhoods belong to a sub-district). Demographic structures among the three sub-districts are similar in terms of age, gender, migrant population, and Han/non-Han nationality. Educational levels, usually in line with economic levels, are also similar. Reflecting the current Chinese situation, in all areas the average household consists of around 2.5 persons (Table 3). 

**Figure 3:**
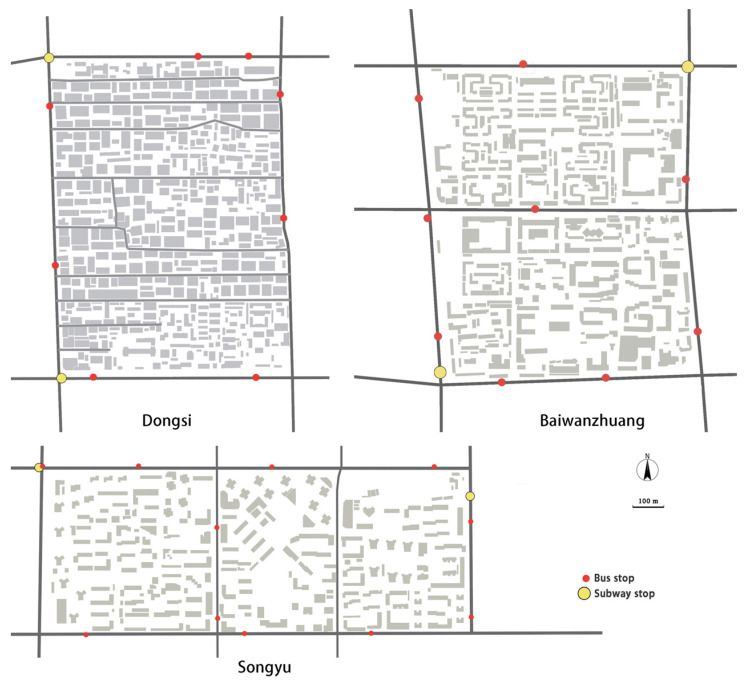
Bus and subway stops in the neighborhoods.

Further, while conducting the urban analysis, we also found similarities in the districts’ public transportation systems (Figure 3). The three studied neighborhoods have two subway stations at the corners, and about 10 bus stops in the surrounding area in each neighborhood. The Dongsi neighborhood has relatively few bus stops (8), and the Songyu neighborhood has the most (11). However, considering that sidewalk connectivity was cut by the main roads in Songyu and Baiwanzhuang, the three areas have similar accessibility. Further, it may be noted that in each neighborhood the subway stations and bus stop locations are evenly distributed over the neighborhood, to ensure proximity to public transportation for all the residents.

## 5. Results

The three neighborhoods are appropriate to analyze how built environment promotes PA, given their differences in the built environment and the similarity in the social economic status. We assessed the built environments of three neighborhoods in Beijing (each selected as representatives of a specific neighborhood type) by adopting an urban analysis method to assess the built environment, and measured the residents’ PA level and their perception of the neighborhoods with questionnaires. Then the PA levels in the three neighborhoods are compared and linked to the urban analysis maps and residents’ perceptions of BE. The following subsection will provide detailed data analyses of these assessments.

### 5.1. Built Environment Analysis

#### 5.1.1. Street Connectivity

**Figure 4:**
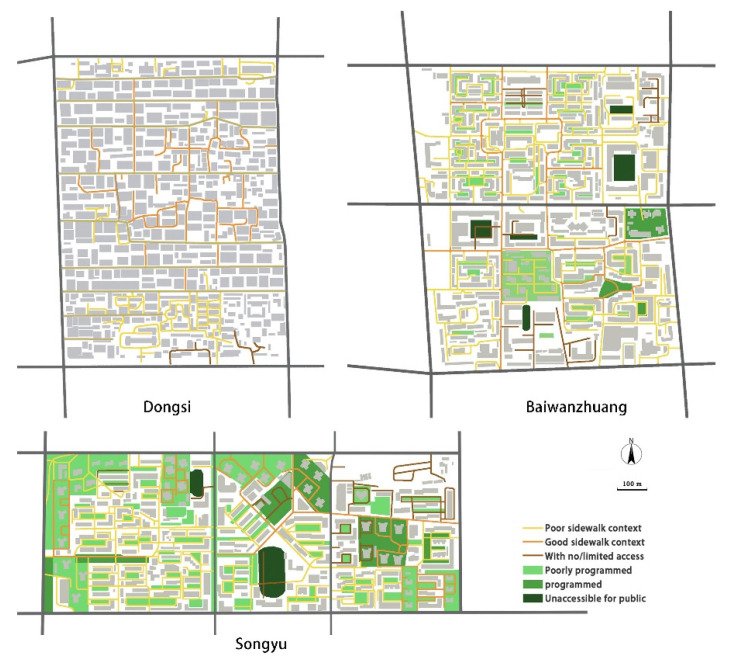
Sidewalks and green structures in the neighborhoods.

For physical activity levels, abundance and quality of sidewalks are key. Figure 4 indicates that the sidewalks occur in well-connected webs in Baiwanzhuang and Songyu neighborhoods, but less so in Dongsi, when we would focus on the number of cross-sections (the three-leg sidewalk intersections are 75, 153, 167 per square kilometer for Dongsi, Baiwanzhuang, and Songyu, respectively). In the Dongsi neighborhood, the traffic structure is simple and clear-seven east–west straight roads go through the neighborhood, and several north–south alleys connect the roads. The narrowness of the alleys makes it difficult for cars to go through them, thus making the north–south alleys pedestrian- and cyclist-friendly. Furthermore, the clear east–west road structure enhances accessibility to facilities such as shops and restaurants, despite the fact they are located mainly along the boundaries of the neighborhood.

Based on the community-centered concept, Baiwanzhuang and Songyu were constructed as pedestrian friendly neighborhoods, with sidewalks in between buildings and public areas, and a variety of choices provided to reach a destination. However, using field observation we noticed that in the Baiwanzhuang and Songyu neighborhoods many sidewalks were inaccessible or had limited access times. Additionally, in these two neighborhoods the city’s main road(s) cut the neighborhoods into several sub-neighborhoods. This indicates that connectivity was somehow reduced within these neighborhoods.

**Figure 5:**
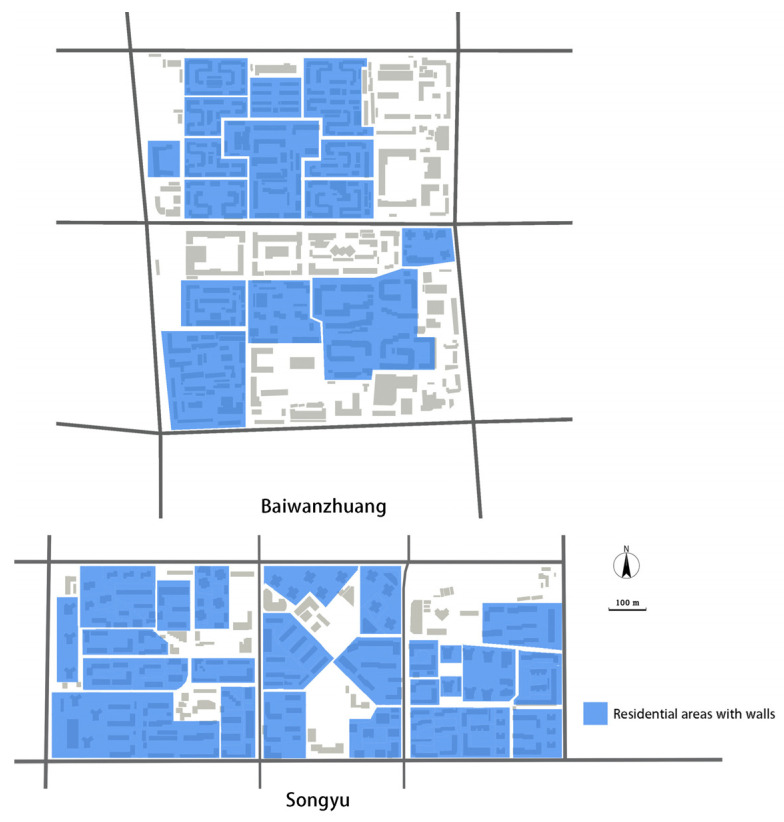
Residential area zones with walls in the neighborhoods.

Gated living areas exacerbate the divisions. Dongsi is an open-block neighborhood with no walls. Baiwanzhuang and Songyu are different. They are gated neighborhoods (Figure 5); many residential areas within the neighborhoods are surrounded by walls, sometimes with access control. As shown in Figure 4, both neighborhoods had more than 10 gated residential areas, all of them surrounded by walls and (limited access) gates, which form great barriers for using sidewalks. The walls give outsiders an unwelcoming impression, thereby making the inside facilities within that area, including the sidewalks, almost unusable for people not living there. Another issue is that residential areas equipped with access control sometimes require access cards to pass, or they may be completely closed at certain times, such as late at night. In some extreme cases the gates are always locked.

One problem common to all three neighborhoods is that parked cars block the sidewalks. This can make them seem unpleasant and unsafe for walking. In the last two decades in China, for a family to own a car has become more and more popular, but when the three studied neighborhoods were built, parking was not taken into consideration. Now the problem has arisen, and many public areas, including sidewalks, are occupied by private cars. This leads to the poor quality of sidewalks as shown in the maps.

#### 5.1.2. Residential Density and Land-Use Diversity

**Figure 6:**
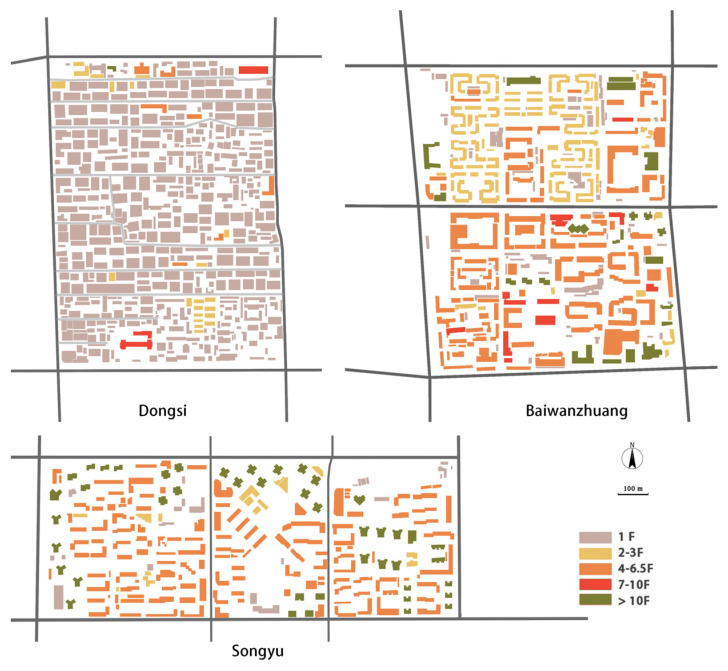
Building heights in the neighborhoods.

From the perspective of residential density, the historical neighborhood of Dongsi has a low density (14,943 people per square kilometer for Dongsi, and 30,392, 33,333 for Baiwanzhuang and Songyu respectively), and is therefore disadvantageous for walking and cycling (according to Table 1). Baiwanzhuang has mainly 3- to 5-floor residential buildings built in the 1950s, and 6-floor buildings built in the 1970s to 1980s. Songyu is a neighborhood built in the 1990s, and contains residential buildings of 5 to 7 floors, together with tower buildings of 16 to 20 floors. In the latter two neighborhoods, the dwelling units are usually 30 to 40 square meters, which is quite standard for the time they were built. Unlike these two neighborhoods, most residential buildings in the Dongsi neighborhood are Siheyuan, where the housing unit is large in area but with only a ground floor (Figure 6).

**Figure 7:**
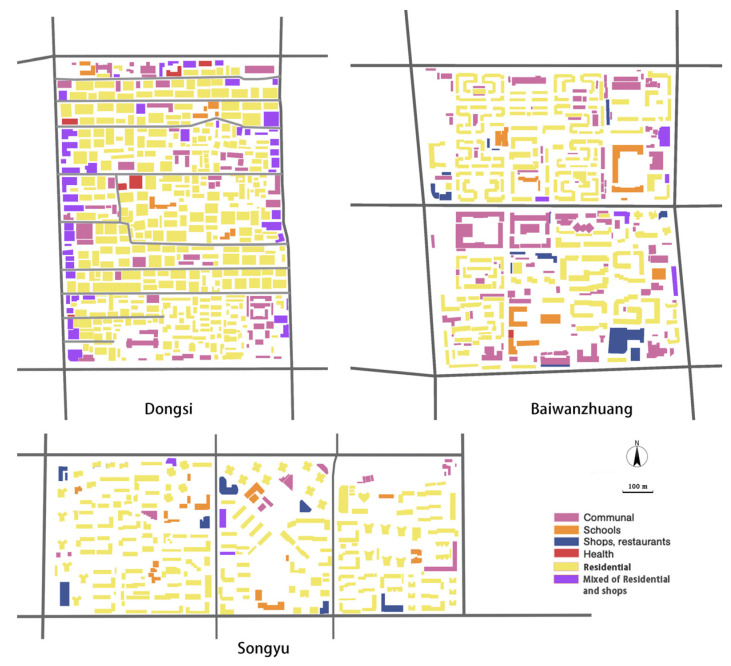
Building types in the neighborhoods.

As for land-use diversity, the main function of these three neighborhoods is residential. Other than residential buildings, all of the neighborhoods have shops, restaurants, schools and communal buildings, but with different distribution patterns. Taking shops and restaurants as examples: in Dongsi neighborhood, most shops and restaurants are distributed throughout the area, with a few located centrally. Even though they may seem to lack accessibility, thanks to the area’s straight street patterns the shops serve the entire neighborhood well. Fewer shops and restaurants are available in Baiwanzhuang, and these are distributed unevenly; the Figure 7 shows that the limited number and uneven distribution of shops cannot serve the whole neighborhood. Moreover, the largest shopping mall in the south is no longer in use. In contrast, in spite of being separated by main roads, Songyu’s three sub-neighborhoods have adequate and well-distributed shops and restaurants. Moreover, within these sub-neighborhoods, residents living in the center as well as the fringe areas all have shops nearby.

#### 5.1.3. Green Space Availability

Most green areas were observed in Songyu among the three neighborhoods. In the center of Songyu is a neighborhood park, and between many buildings are green areas that reflect how green space was valued in neighborhood design at that time. However, when taking the nature, accessibility, and quality of these green spaces into account, Songyu does not rate high for green spaces. First, many green spaces contain bushes or are poorly managed, which results in lower-quality green areas. Second, accessibility to many of the green areas is poor. As shown in Figure 4, the center park has only limited access time, during both day and evening. Moreover, the green in the gated residential areas also provides limited access. Qualitatively speaking, well-programmed green areas are in the minority; most green areas are poor programmed or non-managed.

In the Baiwanzhuang neighborhood, green spaces have different characteristics in the north and south. The northern part has more buildings from the 1950s and a more precise spatial structure. In the north, green can usually be found between buildings and in front and back yards. However, the green spaces between residential buildings are poorly programmed, and consist mainly of trees. The south also has green spaces between buildings; these consist of bushes and grass, and are somewhat better programmed. Moreover, in the south-east, there is a well-programmed public green space with no access control. However, because divided by the main road, the green spaces in the north and south cannot easily be shared by each other’s residents. In the Dongsi neighborhood no open green space is available.

### 5.2. Self-Reported PA and Perceptions

In total, 519 questionnaires were collected in the three neighborhoods, of which 435 were considered to be valid for analysis (the other 84 were excluded because of too many missing or false values). This left the efficiency rate of the responses at 83.8%. We had 128, 147, and 160 valid questionnaires for Dongsi, Baiwanzhuang, and Songyu, respectively.

There were more female than male respondents in three neighborhoods. In Songyu, the gender difference was most obvious, with 65.0% of responses from females. The rate was relatively lower in the Dongsi neighborhood (55.8%). A similar demographic structure was shown in respondents’ original provinces/cities—in each studied neighborhood, more than 3/4 were local Beijing residents. Furthermore, the socio-economic status in terms of education and economic level was similar in the three neighborhoods (Table 4). In 2020, Beijing’s average net monthly household income is around CNY 14,500 if considering the average 2.5 person/household [40]. Our results show that the average incomes in the three neighborhoods are below the city average but similar with each other. The average educational level and the average age were the same in the three neighborhoods.

The question ‘Do you want to move to another neighborhood in the following 6-months?’ was to explore the impact caused by self-selection bias. In total, 18.0%, 5.8%, and 11.3% residents answered ‘Yes’ in the Dongsi, Baiwanzhuang, and Songyu neighborhoods, respectively. Which showed that most people were satisfied with their residential neighborhoods.

#### 5.2.1. Activity Duration

**Figure 8:**
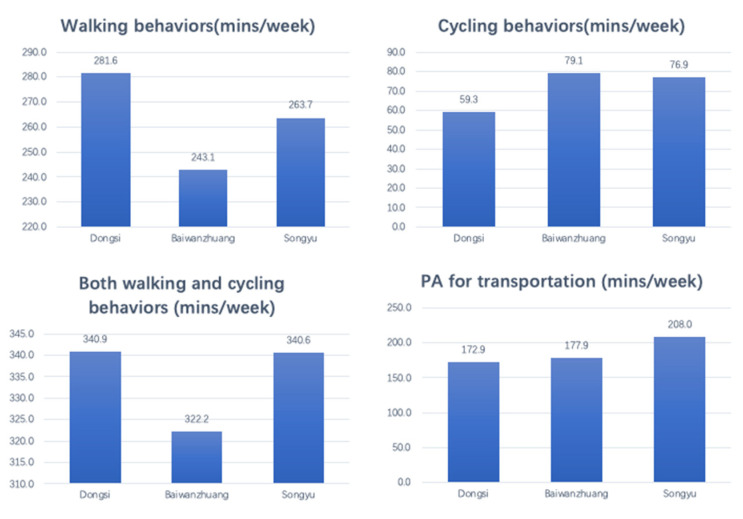
Physical Activities Durations in a general week in three neighborhoods.

More walking behavior took place in Dongsi (281.6 min/week), followed by Songyu and Baiwanzhuang with 263.7 and 243.1 min/week (Figure 8). Cycling duration was similar in Baiwanzhuang and Songyu, and least cycling occurred in Dongsi (76.9, 79.1 and 59.3 min/week). 

As walking and cycling supplement each other, a combination of the two (PA) presents a complete picture (Figure 8). PA duration in a general week counted 340.9 and 340.6 min/week in Dongsi and Songyu. Baiwanzhuang counted the least 322.2 min/week. Most transportation-related PA (e.g., work, shopping) were presented in Songyu.

#### 5.2.2. Activity Frequency

Physical activity behavior was divided into categories related to people’s motivation/destination, namely: (1) school and work; (2) shopping; (3) other errands (banks, restaurants); (4) recreation. The first three categories were travel-related PA, and the last category was recreational PA. 

**Figure 9:**
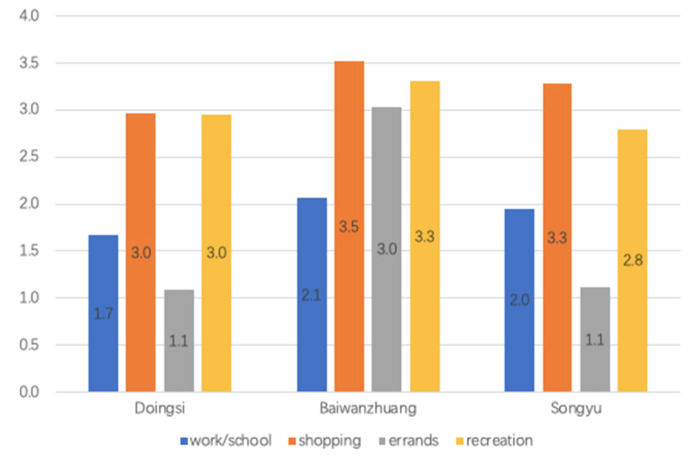
The amount of days people conduct PA in a general week in three neighborhoods.

On average, how many days in a week people conducted PA (including walking and cycling) according to motivation categories was used to measure activity frequency (Figure 9). In Baiwanzhuang, the average days for each category of PA motivation were high. We found higher transportation-related PA frequency in Songyu than Dongsi. On the other hand, lowest recreational PA frequency was found in Songyu. The highest PA motivation category in all three neighborhoods was go/back for shopping which motived 3.0 to 3.5 days of PA, followed by go/back for recreation (2.8 to 3.3 days). 

#### 5.2.3. Neighborhood Perceptions

A 5-point Likert scale was applied in questions to investigate residents’ perception of the neighborhood environments (1: very unsatisfied/very bad, 2: unsatisfied/bad, 3: neutral, 4: satisfied/good, 5: very satisfied/very good). The questions pertained to (1) green and playground areas, (2) sidewalks, (3) safety, and (4) aesthetics. 

The satisfaction with the green and playground areas was higher in Songyu (3.3 on average) than Baiwanzhuang (2.9). More than half of the residents (58.0%) did not hold strong opinions regarding the green and playground areas (neutral) in Baiwanzhuang, while the more diverse result was shown for Songyu. No data is shown for Dongsi because there were no green spaces. More than half of the residents were satisfied with the sidewalks (responded ‘good/very good’) in Dongsi (55.0%) and Songyu (52.5%), and the satisfaction rate was 48.2% in Baiwanzhuang. Around 1/5 were not satisfied with the sidewalks in all three neighborhoods (Table 5).

In general, the residents felt safe living in the three neighborhoods (more than 80% perceived their neighborhood as safe/very). The average scores of perceived safety were the highest in the four measured perceptions for every neighborhood, which were all above 4.0. Namely, 4.4, 4.1, and 4.2 for Dongsi, Baiwanzhuang, and Songyu, respectively. An obvious lower average rate of the neighborhood’s aesthetics (3.0) was found in Baiwanzhuang, compared to the other two neighborhoods (3.5 and 3.4 for Dongsi and Baiwauzhuang). Similar results showed when looking at the percentages of people who perceive their living neighborhood environment as ‘very nice/nice’: 47.5%, 34.5%, and 41.3% for Dongsi, Baiwanzhuang, and Songyu, respectively. 

In sum, our data show the pattern as indicated in Table 6.

## 6. Discussion and Conclusions

By comparing three neighborhoods in Beijing, China, we found confirmation for our assumption that parameters that affect physical activity and therefore health (1) work in concert and must be studied in a comprehensive way, and (2) the often-used parameters (density, road connectivity, etc.) can have comparable values while concealing vital differences that radically change behavioral effects. 

Our data show that the impacting dimensions clearly have to be considered in concert. One feature appears to be able to compensate for the lack of another. Treated separately, individual neighborhood characteristics lead to counterintuitive evidence. Dongsi showed the highest walking times while also showed the lowest density, land-use diversity and green space availability. Based on these parameters alone, Dongsi would be expected to have low activity levels and in need of improvement. Apparently, the well-appreciated safety, sidewalks, and easy access from homes to the street make people walk relatively much. This would falsify the popular thesis that density, green space and diversity induce more PA. Additionally, the lowest walking and overall PA (Baiwanzhuang) happens in a neighborhood with the best road connectivity, that appears to be hampered by the quality of the sidewalks and the low aesthetics perception. 

Apparently, different aspects clearly work as a totality [46]. Their interaction with each other influences residents’ behaviors. Large amounts and good quality of green areas in a neighborhood can be beneficial in promoting PA. However, the actual use of the green areas might be reduced because of poor accessibility caused by damaged sidewalks or neighborhood access controls. The whole impacting mechanism is complex: the quality of sidewalks and green spaces both impact the environment aesthetics. 

Secondly, features of the built environment must be measured in a nuanced way that properly reflects their influence on lifestyle choices of residents. Our method showed that the *perceived* quality of sidewalks is better predictors of walking than physical and objective connectivity of the road network. In Baiwanzhuang, the well-connected streets received a low rating by the residents; field observation brought many usability problems to light. The low rating explains why walking behavior there is much lower than expected based on a calculated road connectivity parameter alone. In addition, neighborhoods may be of similar calculated population density, but in Dongsi this in fact is a maze of streets with low houses whereas Songyu has wide streets with multi story apartment blocks spaced more widely. The PA behavior happens much more naturally in the first compared to the latter. This logical difference is concealed by a mere calculated density figure. 

A holistic and human-centered approach is essential in assessing the neighborhood environment and its actual influence on physical activity of residents. Higher density/land-use mix, street connectivity and public transport availability only lead to more PA when they are combined, and when residents are positive about the quality for their use experience. The importance of how residents experience the environment is evident in Songyu: the road connectivity and density/diversity should be beneficial for PA, but its residents did not participate the most in PA. Although there were many sidewalks, the poor quality apparently became a barrier to frequent PA. Similarly, green areas require to sufficient in both quantity and quality in order to actually promote PA [21]. Quantity-wise, Songyu is the greenest neighborhood and equipped with a park. However, field observation showed that poor management of the green areas has not helped to promote residents’ physical activities. Many green spaces were occupied by cars, making them unusable for residents; others were overgrown or surrounded by fences. A key point is their management: some green areas attracted mosquitoes and insects, and had a bad smell. Residents were more satisfied with the green areas in Songyu than in Baiwanzhuang. However, we argue that this advantage is brought by the quantity. With a better quality of green spaces, the advantage should be even greater. It is important for policymakers to understand that besides designing for a healthy neighborhood, managing the built and green environment is just as important.

Aesthetics and safety of public spaces are key requirements for PA. Dongsi rates highest in both perceived safety and neighborhood aesthetics, while Baiwanzhuang rates lowest. Previous evidence suggested that these two dimensions have only a limited positive relation to PA. In our study, however, they correspond with the duration of PA in the three neighborhoods. Although Dongsi is the only open block neighborhood, residents perceived it as safe. Additionally, even though it provides fewer facilities such as green spaces, the neighborhood is perceived as nice. The simple and clear spatial pattern appears to enhance people’s perception of aesthetics and safety.

A designer and policy-maker should also be aware of the history and development of a neighborhood [47] when interpreting feature of the built environment. For example, in the traditional Beijing neighborhood Dongsi, one dwelling unit is the Siheyuan. Historically, at the time the neighborhood was built people lived together in large families, thus dwelling units were built large to accommodate such families. However, in line with social developments, nowadays most people no longer live in large families (the average family unit is two to three persons), and sharing dwelling units has become normal in the Dongsi neighborhood. In some cases, more than 10 households (not families/relatives) share one Siheyuan for financial and other reasons. The other two neighborhoods were built after 1949 (the year of the establishment of the People’s Republic of China); because people started living in smaller households, the dwelling unit became much smaller in area. If assessing the residential density without understanding the historical development in how people live there, the calculated indicator may be deviate from social reality. 

More research is needed to further develop this understanding. Due to limited access to digital spatial data, the urban analyses were mostly mapped by hand and with the use of the Baidu Map, there may therefore be some discrepancies, especially in the areas with strict access controls. In this paper we focus mostly on the environment within the neighborhoods. It must be noted that adjoining neighborhoods and further attractions also have an impact. For future studies, a research method that treats influential dimensions of the built environment as a whole, while valuing the aspects of both quantity and quality is required. Moreover, more insights from the perspective of urban planning/design should be brought in to assess the built environment in order to create healthier neighborhoods. 

## Figures and Tables

**Table 1:** Built environment parameters, expected impacts on PA, and operational methods.

Logic	Studies Based on Generic Spatial Data	Holistic User-Focused Studies
Density and land-use diversity: High population density enables diversity of services near people’s homes, enabling residents to walk to shops, work, etc.	Define density as the number of dwellings per hectare; land-use types calculated into an entropy index	Considers how dwellings are divided over the area and residents’ experience: shops not equally valued by local residents according to the location etc.
Street connectivity: Physical activity requires availability of a network of publicly accessible streets to allow a variety of possible walks	Use street patterns from maps and treat all connections as equal	Conducts analysis of qualities of streets, including narrow alleys; also assesses usability of sidewalks
Green space: Availability of green space enables people to relax or to do exercise outdoors	Consider physical number/surface of green space; assume all green spaces are equally valuable for residents	Takes into account nature, accessibility and quality of green spaces
Public transport: When features mentioned above are present but longer trips require a car, less walking and cycling will take place	Density of bus and subway stops	Not only the number of stops but also their distribution and services
Esthetics: Motivating people to go outside	Mostly self-reported data	Self-reported data and sidewalk context
Safety: Not be barriers for going outside	Mostly self-reported data	Perceived safety

**Table 2:** Neighborhood typologies in China.

Neighborhood Typology	Period	Planning Strategies
Ancient China	To 19th century	Follows traditional Chinese urban planning theory; urban areas are symmetrical, square, with a (narrow) alley between buildings
Post-war	1940s–1978	Follow ‘neighborhood unit’ and ‘neighborhood areas’ design concept. Facilities, like schools, included in one neighborhood to create community-centric lifestyle
Post-cultural revolution	1978–1998	Uniform planning, design, construction and management. Residential areas combined with 4–6 floor buildings and tower buildings; green spaces and public areas are valued in design
Contemporary	1998–now	Market-oriented neighborhood development; diversity in neighborhood construction; mostly gated

**Table 3:** The socio-demographic data of the three sub-districts.

	Dongsi Sub-District (Includes Dongsi Neighborhood)	Zhanlan Street Sub-District (Includes Baiwanzhuang Neighborhood)	Panjiayuan Sub-District (Includes Songyu Neighborhood)
Total number of residents	113,115	43,731	130,925
Age under 20(%)	13.77%	13.15%	9.42%
Age above/equals 65(%)	10.91%	13.57%	14.78%
Female(%)	51.55%	50.30%	50.85%
Illiteracy(%)	1.21%	1.46%	1.45%
Education level above/equals high school(%)	65.51%	68.16%	67.67%
Migrant population(%)	24.58%	27.61%	23.52%
Han nationality(%)	92.29%	95.20%	95.11%
Average household size (person/household)	2.56	2.54	2.39

Data resource [39]:Office for the Sixth population census of Beijing municipality et al., 2010.

**Table 4:** Socio-economic status of the three neighborhoods.

	Dongsi	Baiwanzhuang	Songyu
Valid questionnaires (number)	128	147	160
Female (%)	61.7	55.8	65.0
Local Beijing residents (%)	78.1	75.5	76.9
Average age group (years old)	40–49	40–49	40–49
Average Education level	High school	High school	High school
Average Household net monthly income level (Yuan)	6000–10,000	6000–10,000	6000–10,000

**Table 5:** Satisfaction of the neighborhoods.

		***1***	***2***	***3***	***4***	***5***	***Average***
***Green spaces***	Dongsi	/	/	/	/	/	/
	Baiwanzhuang	14.8%	8.6%	58.0%	11.1%	7.4%	2.9
	Songyu	8.8%	12.5%	36.3%	25.0%	17.5%	3.3
***Sidewalks***	Dongsi	10.0%	9.2%	25.8%	33.3%	21.7%	3.5
	Baiwanzhuang	4.9%	17.3%	29.6%	38.3%	9.9%	3.3
	Songyu	10.0%	11.3%	26.3%	30.0%	22.5%	3.4
***Safety***	Dongsi	0.0%	0.8%	5.0%	50.8%	43.3%	4.4
	Baiwanzhuang	4.9%	0.0%	12.4%	50.6%	32.1%	4.1
	Songyu	1.3%	1.3%	10.0%	48.8%	38.8%	4.2
***Aesthetics***	Dongsi	6.7%	5.8%	40.0%	28.3%	19.2%	3.5
	Baiwanzhuang	12.4%	22.2%	30.9%	25.9%	8.6%	3.0
	Songyu	6.3%	3.8%	48.8%	27.5%	13.8%	3.4

**Table 6:** Comparison of main built environment dimensions and PA levels in three neighborhoods.

		Dongsi	Baiwanzhuang	Songyu
(A) Urban-analysis	Street connectivity	Fair connectivity. Pedestrian-friendly design. 75 three leg sidewalk intersections/km^2^.	Good connectivity in general, but blocked by factors such as cars and walls. 153 three leg sidewalk intersections/km^2^.	Good connectivity in general, but also somewhat blocked. 167 three leg sidewalk intersections/km^2^.
	Density and land-use diversity	Relatively Low; Population density: 14,943 person/km^2^.	Medium; Population density: 30,392 person/km^2^.	Relatively High; Population density: 33,333 person/km^2^.
	Green space	Non-existent	Middle level quantity and low quality	High in quantity but low in quality
	Public transport availability	Good; 11 bus and subway stops/km^2^	Good; 11 bus and subway stops/km^2^	Good; 12 bus and subway stops/km^2^
(B) Perception about public spaces	Green spaces (Likert average)	/	2.9	3.3
	Sidewalks (Likert average)	3.5	3.3	3.4
	Safety (Likert average)	4.4	4.1	4.2
	Aesthetics (Likert average)	3.5	3.0	3.4
(C) Activity levels	Walking duration (mins/week)	Mean: 281.6 SD: 283.1	Mean: 243.1 SD: 244.7	Mean: 263.7 SD: 267.6
	Cycling duration (mins/week)	Mean: 59.3 SD: 119.9	Mean: 79.2 SD: 117.9	Mean: 76.9 SD: 142.2
	Total duration (mins/week)	Mean: 340.9 SD: 304.0	Mean: 322.2 SD: 269.4	Mean: 340.6 SD: 314.2
	Trip motive (frequency in a week)	Shopping > recreation > work/school > errands	Shopping > recreation > errands > work/school	Shopping > recreation > work/school > errands

## Data Availability

Data available on request due to ethical restrictions.

## References

[1] United Nations (2018). Revision of World Urbanization Prospects.

[2] (2021). World Health Organization; Urban Health. https://www.who.int/health-topics/urban-health.

[3] World Health Organization (2013). Global Action Plan for the Prevention and Control of NCDs 2013–2020. https://apps.who.int/iris/handle/10665/94384.

[4] World Health Organization (2018). Noncommunicable Diseases (NCD) Country Profiles. https://apps.who.int/iris/handle/10665/274512.

[5] Barton H., Tsourou C. (2013). Healthy Urban Planning.

[6] World Health Organization (2019). A Multilevel Governance Approach to Preventing and Managing Noncommunicable Diseases: The Role of Cities and Urban Settings. WHO European High-Level Conference on Noncommunicable Diseases. https://www.euro.who.int/en/health-topics/environment-and-health/urban-health/publications/2019/a-multilevel-governance-approach-to-preventing-and-managing-noncommunicable-diseases-the-role-of-cities-and-urban-settings-2019.

[7] Mckeown T. (1979). The Role of Medicine: Dream, Mirage or Nemesis?.

[8] World Health Organization (1994). WHO Healthy Cities: A Program Framework, A Review of the Operation and Future Development of the WHO Healthy Cities Program.

[9] Handy S.L., Boarnet M.G., Ewing R., Killingsworth R.E. (2002). How the built environment affects physical activity: Views from urban planning. Am. J. Prev. Med..

[10] Southworth M. (2005). Designing the walkable city. J. Urban Plan. Dev..

[11] Saelens B.E., Handy S.L. (2008). Built environment correlates of walking: A review. Med. Sci. Sports Exerc..

[12] Fonseca F., Ribeiro P.J.G., Conticelli E., Jabbari M., Papageorgiou G., Tondelli S., Ramos R.A.R. (2021). Built environment attributes and their influence on walkability. Int. J. Sustain. Transp..

[13] Roof K., Oleru N. (2008). Public health: Seattle and King County’s push for the built environment. J. Environ. Health.

[14] World Health Organization (2022). Physical Activity. https://www.who.int/health-topics/physical-activity.

[15] World Health Organization (2019). Global Action Plan on Physical Activity 2018–2030: More Active People for a Healthier World. https://apps.who.int/iris/bitstream/handle/10665/272722/9789241514187-eng.pdf.

[16] Song S., Yap W., Hou Y., Yuen B. (2020). Neighbourhood built Environment, physical activity, and physical health among older adults in Singapore: A simultaneous equations approach. J. Transp. Health.

[17] Clary C., Lewis D., Limb E., Nightingale C.M., Ram B., Page A.S., Cooper A.R., Ellaway A., Giles-Corti B., Whincup P.H. (2020). Longitudinal impact of changes in the residential built environment on physical activity: Findings from the ENABLE London cohort study. Int. J. Behav. Nutr. Phys. Act..

[18] Durand C.P., Andalib M., Dunton G.F., Wolch J., Pentz M.A. (2011). A systematic review of built environment factors related to physical activity and obesity risk: Implications for smart growth urban planning. Obes. Rev..

[19] Wang S., Liu Y., Lam J., Kwan M.-P. (2021). The effects of the built environment on the general health, physical activity and obesity of adults in Queensland, Australia. Spat. Spatio-temporal Epidemiol..

[20] Astell-Burt T., Feng X., Kolt G.S. (2014). Green space is associated with walking and moderate-to-vigorous physical activity (MVPA) in middle-to-older-aged adults: Findings from 203 883 Australians in the 45 and Up Study. Br. J. Sports Med..

[21] Zhang Y., Van den Berg A.E., Van Dijk T., Weitkamp G. (2017). Quality over Quantity: Contribution of Urban Green Space to Neighborhood Satisfaction. Int. J. Environ. Res. Public Health.

[22] Shaked H., Schechter C. (2017). Definitions and development of systems thinking. Systems Thinking for School Leaders.

[23] United States Agency for International Development Human-Centered Design. https://www.usaid.gov/cii/human-centered-design.

[24] Salvo D., Reis R.S., Stein A.D., Rivera J., Martorell R., Pratt M. (2014). Characteristics of the Built Environment in Relation to Objectively Measured Physical Activity Among Mexican Adults, 2011. Prev. Chronic Dis..

[25] Zhao P., Wan J. (2020). Examining the effects of neighbourhood design on walking in growing megacity. Transp. Res. Part D: Transp. Environ..

[26] Duncan M.J., Winkler E., Sugiyama T., Cerin E., duToit L., Leslie E., Owen N. (2010). Relationships of Land Use Mix with Walking for Transport: Do Land Uses and Geographical Scale Matter?. J. Urban Health.

[27] Ewing R., Cervero R. (2010). Travel and the built environment: A meta-analysis. J. Am. Plan. Assoc..

[28] Lu Y., Chen L., Yang Y., Gou Z. (2018). The Association of Built Environment and Physical Activity in Older Adults: Using a Citywide Public Housing Scheme to Reduce Residential Self-Selection Bias. Int. J. Environ. Res. Public Health.

[29] Wang H., Dai X., Wu J., Wu X., Nie X. (2019). Influence of urban green open space on residents’ physical activity in China. BMC Public Health.

[30] Wang Z., Qin Z., He J., Ma Y., Ye Q., Xiong Y., Xu F. (2019). The Association between Residential Density and Physical Activity among Urban Adults in Regional China. BMC Public Health.

[31] Frank L.D., Saelens B., Powell K.E., Chapman J.E. (2007). Stepping towards causation: Do built environments or neighborhood and travel preferences explain physical activity, driving, and obesity?. Soc. Sci. Med..

[32] Sundquist K., Eriksson U., Kawakami N., Skog L., Ohlsson H., Arvidsson D. (2011). Neighborhood walkability, physical activity, and walking behavior: The Swedish Neighborhood and Physical Activity (SNAP) study. Soc. Sci. Med..

[33] Kikuchi H., Nakaya T., Hanibuchi T., Fukushima N., Amagasa S., Oka K., Sallis J.F., Inoue S. (2018). Objectively Measured Neighborhood Walkability and Change in Physical Activity in Older Japanese Adults: A Five-Year Cohort Study. Int. J. Environ. Res. Public Health.

[34] Frank L.D., Sallis J.F., Saelens B.E., Leary L., Cain K., Conway T.L., Hess P.M. (2010). The development of a walkability index: Application to the Neighborhood Quality of Life Study. Br. J. Sports Med..

[35] Zapata-Diomedi B., Veerman J.L. (2016). The Association between Built Environment Features and Physical Activity in the Australian Context: A Synthesis of the Literature. BMC Public Health.

[36] Bailey E.J., Malecki K.C., Engelman C.D., Walsh M.C., Bersch A.J., Martinez-Donate A.P., Peppard P.E., Nieto F.J. (2014). Predictors of discordance between perceived and objective neighborhood data. Ann. Epidemiol..

[37] Porter A.K., Kohl H.W., Perez A., Reininger B., Gabriel K.P., Salvo D. (2018). Perceived social and built environment correlates of transportation and recreation-only bicycling among adults. Prev. Chronic Dis..

[38] Saelens B.E., Sallis J.F., Black J.B., Chen D. (2003). Neighborhood-based differences in physical activity: An environment scale evaluation. Am. J. Public Health.

[39] Office for the Sixth Population Census of Beijing Municipality, Beijing Municipality Bureau of Statistics, Survey Office of the National Bureau of Statistics in Beijing (2010). Tabulation on the 2010 Population Census of Beijing Municipality (Town and Sub-District Volume).

[40] (2021). Beijing Bureau of Statistics. http://tjj.beijing.gov.cn/tjsj_31433/yjdsj_31440/jmsz_32036/2020/202101/t20210120_2227744.html.

[41] Zhao W., Kai Y. (2009). 60 years of community planning in China. Des. Community.

[42] Clifton K.J., Smith A.D.L., Rodriguez D. (2007). The development and testing of an audit for the pedestrian environment. Landsc. Urban Plan..

[43] Kaczynski A.T., Stanis S.A.W., Besenyi G.M. (2012). Development and Testing of a Community Stakeholder Park Audit Tool. Am. J. Prev. Med..

[44] Craig C.L., Marshall A.L., Sjöström M., Bauman A.E., Booth M.L., Ainsworth B.E., Pratt M., Ekelund U.L., Yngve A., Sallis J.F. (2003). International physical activity questionnaire: 12-country reliability and validity. Med. Sci. Sports Exerc..

[45] Boone-Heinonen J., Guilkey D.K., Evenson K.R., Gordon-Larsen P. (2010). Residential self-selection bias in the estimation of built environment effects on physical activity between adolescence and young adulthood. Int. J. Behav. Nutr. Phys. Act..

[46] Barton H., Grant M., Guise R. (2021). Shaping Neighbourhoods: For Local Health and Global Sustainability.

[47] Wagenaar C. (2015). Town Planning in the Netherlands since 1800: Responses to Enlightenment Ideas and Geopolitical Realities.

